# Novel bimodal TRBD1-TRBD2 rearrangements with dual or absent D-region contribute to TRB V-(D)-J combinatorial diversity

**DOI:** 10.3389/fimmu.2023.1245175

**Published:** 2023-09-07

**Authors:** Anastasia O. Smirnova, Anna M. Miroshnichenkova, Laima D. Belyaeva, Ilya V. Kelmanson, Yuri B. Lebedev, Ilgar Z. Mamedov, Dmitriy M. Chudakov, Alexander Y. Komkov

**Affiliations:** ^1^ Center for Molecular and Cellular Biology, Skolkovo Institute of Science and Technology, Moscow, Russia; ^2^ Genomics of Adaptive Immunity Department, Shemyakin-Ovchinnikov Institute of Bioorganic Chemistry, Moscow, Russia; ^3^ Abu Dhabi Stem Cells Center (ADSCC), Abu Dhabi, United Arab Emirates; ^4^ Department of Biomolecular Sciences and Department of Molecular Neuroscience, Weizmann Institute of Science, Rehovot, Israel; ^5^ Department of Molecular Technologies, Institute of Translational Medicine, Pirogov Russian National Research Medical University, Moscow, Russia; ^6^ Central European Institute of Technology, Masaryk University, Brno, Czechia; ^7^ Dmitry Rogachev National Medical and Research Center of Pediatric Hematology, Oncology, and Immunology, Moscow, Russia

**Keywords:** TRB repertoire, VDJ recombination, NGS - next generation sequencing, T cell, Thymus

## Abstract

T-cell receptor (TR) diversity of the variable domains is generated by recombination of both the alpha (TRA) and beta (TRB) chains. The textbook process of TRB chain production starts with TRBD and TRBJ gene rearrangement, followed by the rearrangement of a TRBV gene to the partially rearranged D-J gene. Unsuccessful V-D-J TRB rearrangements lead to apoptosis of the cell. Here, we performed deep sequencing of the poorly explored pool of partial TRBD1-TRBD2 rearrangements in T-cell genomic DNA. We reconstructed full repertoires of human partial TRBD1-TRBD2 rearrangements using novel sequencing and validated them by detecting V-D-J recombination-specific byproducts: excision circles containing the recombination signal (RS) joint 5’D2-RS – 3’D1-RS. Identified rearrangements were in compliance with the classical 12/23 rule, common for humans, rats, and mice and contained typical V-D-J recombination footprints. Interestingly, we detected a bimodal distribution of D-D junctions indicating two active recombination sites producing long and short D-D rearrangements. Long TRB D-D rearrangements with two D-regions are coding joints D1-D2 remaining classically on the chromosome. The short TRB D-D rearrangements with no D-region are signal joints, the coding joint D1-D2 being excised from the chromosome. They both contribute to the TRB V-(D)-J combinatorial diversity. Indeed, short D-D rearrangements may be followed by direct V-J2 recombination. Long D-D rearrangements may recombine further with J2 and V genes forming partial D1-D2-J2 and then complete V-D1-D2-J2 rearrangement. Productive TRB V-D1-D2-J2 chains are present and expressed in thousands of clones of human antigen-experienced memory T cells proving their capacity for antigen recognition and actual participation in the immune response.

## Introduction

**Figure 1:**
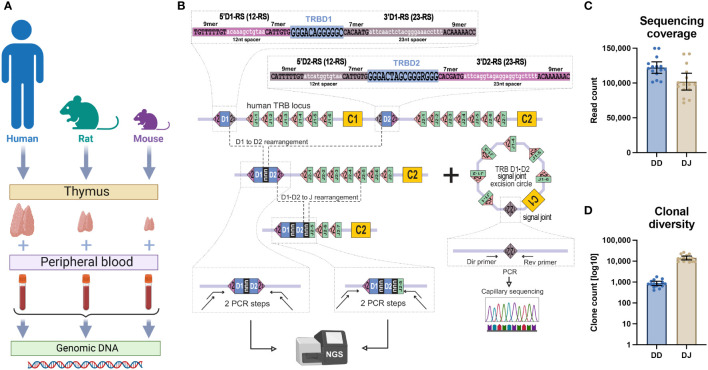
TRB D-D and D-J rearrangement sequencing. **(A)** Type of collected samples. **(B)** PCR-based library preparation and signal joint excision circle detection schemes. **(C)** Obtained sequencing coverage for TRB D-D and TRB D-J libraries. **(D)** Clonal diversity of detected TRB D-D and D-J rearrangements per ~100,000 PBMCs. Bars and whiskers represent the mean with a 95% confidence interval.

T-cell receptor beta (TRB) chains are one of the essential components of the antigen recognition complex in T cells. The power of T-cell receptors (TR) to recognize a wide variety of possible pathogen peptides presented by the major histocompatibility (MH) proteins is provided by the enormous diversity of the variable domains of both alpha (TRA) and beta (TRB) chains (1, 2). This diversity is a result of a somatic process called V-D-J recombination in which variable (V), diversity (D), and joining (J) genes from clusters located in the TRB locus, rearrange together to form complete TRB rearrangement. The V-D-J recombination process is accompanied by the deletion and insertion of a random number of nucleotides in the D-J and V-D junctions. Semirandom choice of each V, D and J gene from the clusters and junctional diversity are the main sources of the required TRB diversity. The classical model of the V-D-J recombination for the TRB locus postulates two sequential stages of rearrangement: D to J joining and subsequent V to D-J joining (3). The final part of TRB chain synthesis is the C gene, which joins V-D-J rearrangement at the RNA level by splicing. The human TRB locus contains 48 functional V genes, two D genes, 13 J genes, and two C genes (1, 4). Importantly, the D, J, and C genes are organized into two distinct clusters downstream of the main pool of TRBV genes. The first group contains TRBD1, TRBJ1-1 to TRBJ1-6, and TRBC1 genes; the second group contains TRBD2, TRBJ2-1 to TRBJ2-7, and TRBC2 genes (**Figure 1**). Each V, D, and J gene is flanked by recombination signal (RS) sequences (1).

There are two canonical types of RS; each type of RS contains a conserved 7-mer and 9-mer separated by a less conservative 12- or 23-nucleotide spacer, forming 12-RS and 23-RS, respectively. Recombination is initiated by binding RAG-1 and RAG-2 recombinase to RS sites, leading to specific DNA cleavage and hairpin generation at the edges of the coding regions (5). Opening of the DNA hairpins is then initiated by the endonuclease DCLRE1C (DNA cross-link repair 1C, Artemis), which makes a single strand cut in a random position near coding regions ends. Exonuclease trims the breaks, and terminal deoxynucleotidyl transferase (TdT) adds several random nucleotides to the single-strand ends produced by DCLRE1C. In the final step polymerase fills the gaps, and ligase IV repairs the breaks. Due to the binding manner of the RAG-1/RAG-2 complex (6), V-D-J rearrangement proceeds solely between RS with different spacer lengths (12/23 rule) (7). The coding region of the V genes (or V-REGION) is flanked at its 3’-end by a V-RS (a 23-RS for TRBV), the coding region of the J genes (or J-REGION) is flanked at its 5’-end by J-RS (a 12-RS for TRBJ), and the coding region of the D genes (D-REGION) is flanked at its 5’-end by a 5’D-RS (12-RS) and at its 3’-end by a 3’D-RS (23-RS) (**Figure 1**). This makes the TRBD1 and TRBD2 genes perfect candidates to recombine with a TRBJ in the 3’ end and a TRBV in the 5’ end but also with each other according to the 12/23 rule. Several sporadic partial TRBD-TRBD rearrangements have indeed been detected previously in mice (8, 9) but have not been validated properly. Rearrangements between D genes have been observed and contribute to the diversity of the TR delta (TRD) chain in humans (10, 11). However, there have been no previous attempts to detect partial TRBD1-TRBD2 rearrangements in human T cells. Even the immunology textbook postulates the impossibility of rearrangements between D genes in the TRB locus (12). Here, using the advances of high-throughput sequencing, we obtained, comprehensively characterized, and validated full repertoires of TRB D-D rearrangements in human, mouse, and rat genomes for the first time.

## Results

### TRB D-D rearrangement detection at the DNA level

Previous studies (13, 14) demonstrated that the conventional concept of V-D-J recombination should be reevaluated based on the actual prevalence rate of noncanonical V-D-D-J rearrangements. Using deep sequencing and an original bioinformatic analysis approach, we explored the hypothesis that TRB containing V-D-D-J rearrangements were the final product of partial TRBD1-TRBD2 rearrangements. To determine whether this genome editing extended beyond humans we performed TRB D-D identification in DNA extracted from human, rat, and mouse thymus and blood cells (**Figure 1A**). Using high-throughput sequencing of amplicons obtained from PCR with primers annealing upstream of 5’D1-RS and downstream of 3’D2-RS (**Figure 1B**), we successfully identified full repertoires of partial TRBD1-TRBD2 genomic rearrangements.

These D-D rearrangements were detected in all DNA samples from thymic cells and peripheral blood mononuclear cells (PBMC), from all three analyzed species (**Figure 1A**). These types of rearrangements were absent in negative control DNA from human CD19+ B cells and nonlymphoid rhabdomyosarcoma cell line.

**Figure 2:**
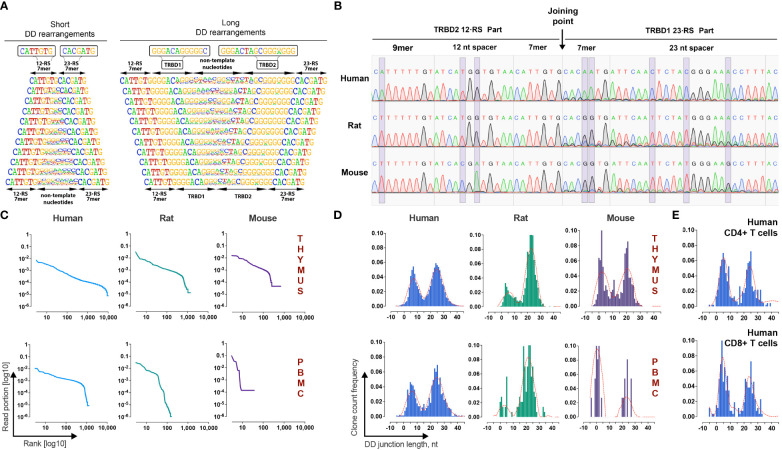
Identification and validation of TRB D-D rearrangements in mammalian lymphocytes. **(A)** Frequency logo diagram for short and long TRB D-D rearrangements detected in human thymus DNA. **(B)** Capillary sequencing of 5’D2-RS - 3’D1-RS signal joints from excision DNA circles from human, rat, and mouse thymi DNA. Violet bands show the position of variable nucleotides across the analyzed species. **(C)** Clonal distributions of TRB D-D rearrangements in human, rat, and mouse thymi and peripheral blood cell DNA. Points on the curve represent unique clonotypes ranked in descending order. The Y-axis value reflects their relative abundance. **(D)** Bimodal distribution of TRB D-D rearrangement junction length in human, rat, and mouse thymi and peripheral blood. **(E)** Bimodal distribution of TRB D-D rearrangement junction length in human helper and cytotoxic T cells. Length calculation performed excluding RS.

In parallel with D-D rearrangements, we additionally identified well-known partial TRB D-J rearrangements in the same human DNA samples. Despite the similar sequencing coverages (**Figure 1C**), the clonal diversity of TRB D-D rearrangements was ten times less than that of D-J rearrangements (**Figure 1D**). However, the absolute number of unique D-D clonotypes (1,000 per 100,000 cells on average) shows that these rearrangements have been subjected to a diversification process during their formation. Typical detected D-D rearrangements represented extremely diverse nucleotide sequences with or without coding D-regions flanked at its 5’ end by the 5’D1-RS and at its 3’ end by the 3’D2-RS (**Figure 2A**). These various junctions contain random nongenomic nucleotide sequences between the TRBD1 and TRBD2 regions in nearly half of the D-D rearrangements detected. The presence of random nontemplated nucleotides and cut D1 and D2 genes are the main source of clonal diversity and, simultaneously, are the hallmark of the V-D-J recombination process.

Additional independent proof that TRB D-D rearrangements are produced by V-D-J recombination machinery is the existence of its specific byproduct – T-cell excision circle DNA that contains the signal joint formed by the 5’D2-RS and the 3’D1-RS. Using PCR with primers specific for 5’D2-RS and 3’D1-RS, we successfully detected 5’D2-RS - 3’D1-RS signal joints in DNA from the thymi of all analyzed species and then confirmed them by capillary sequencing (**Figure 2B**). Sequences with the expected structures containing 5’D2-RS directly connected to 3’D1-RS were detected in the thymic DNA of all three species.

TRB D-D repertoire profiling (**Figure 2C**) shows the presence of TRB D1-D2 rearrangements in both the thymus and PBMC, indicating that T cells released from the thymus still bear partial TRB D-D rearrangements. The repertoires of detected D-D rearrangements are characterized by highly variable clonal abundance. The observed clonal distribution pattern is similar to other TR rearrangements characterized by higher clonal diversity and lower clonal abundance amplitude for the thymus than peripheral blood cells. The observed pattern of clonal variability is in line with other partial TRB rearrangements. Since they are unproductive passenger genomic rearrangements, they change their frequencies following T-cell clonal expansion and contraction in the periphery. Genomic location, cell type specificity, nucleotide structure, signal joint excision circles, and clonal distribution features of detected rearrangements prove that D-D rearrangements are indeed the product of the V-D-J recombination process.

### Bimodal distribution of TRB D-D rearrangements length

A deeper analysis of junction structures of TRB D-D rearrangements shows that their length has a bimodal distribution (**Figure 2D**). Thus, the D-D rearrangements form two groups, “short” with a mean length (excluding RS) of 6 nt and “long” with a mean length of 25 nt. The observed bimodality was characteristic for all analyzed species and for both thymic-derived and PBMC-derived DNA. The same distribution patterns were observed in human helper T cells and cytotoxic T cells (**Figure 2E**). We concluded that both “short” and “long” D-D rearrangements are produced, most likely in the thymus before commitment to the CD4/CD8 lineage, i.e., at the stage of TRB chain synthesis.

**Figure 3:**
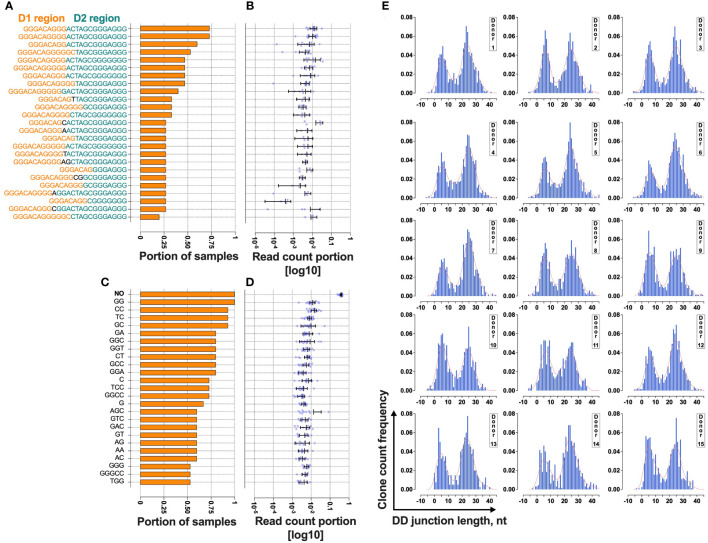
Bimodal distribution of TRB D-D rearrangements in a cross-section of T cells from 15 normal donors. **(A)** The rate of cooccurrence of “long” TRB D- D rearrangements. **(B)** Read count frequencies of the top 25 public “long” D-D clonotypes. **(C)** The rate of cooccurrence of “short” TRB D-D rearrangements. **(D)** Read count frequencies of the top 25 public “short” D-D clonotypes. Bars and whiskers represent the mean with a 95% confidence interval. Black letters in the y-axes represent inserted nucleotides. **(E)** TRB D-D rearrangement length distribution in T cells. Length calculation performed excluding RS.

Next, we analyzed an additional group of DNA samples extracted from the peripheral blood of 15 healthy humans to measure the uniqueness of TRB D-D rearrangements across the human population and the variability of the TRB D-D bimodal distribution (**Figure 3**).

The results show that 25% of “short” TRB D-D rearrangements and 13% of “long” TRB D-D rearrangements are public clonotypes present in two or more different samples (**Figures 3A, C**). This indicates that they have a high generation probability and can be reproduced independently in different individuals. The most frequently shared rearrangements lack inserted nontemplate nucleotides, indicating potential fetal origin (15) due to TdT downregulation at this developmental stage (16). This point is valid for both “short” (**Figure 3B**) and “long” (**Figure 3D**) TRB D-D rearrangements. The “short” D-D rearrangement with zero added nucleotides (perfect signal joint) is the most frequent in each individual occupying 25% of all D-D rearrangement-bearing cells and 38% of cells with “short” D-D rearrangements (**Figure 3D**). Together with a high level of sharing it indicates that this particular rearrangement is not random and is reproduced multiple times in T cells within each individual.

At the same time, the ratio of “short” and “long” TRB D-D can noticeably vary in the blood of different individuals (**Figure 3E**). However, all have the same bimodal distribution pattern.

### Bimodality relates to D-D recombination in two RS sites

**Figure 4:**
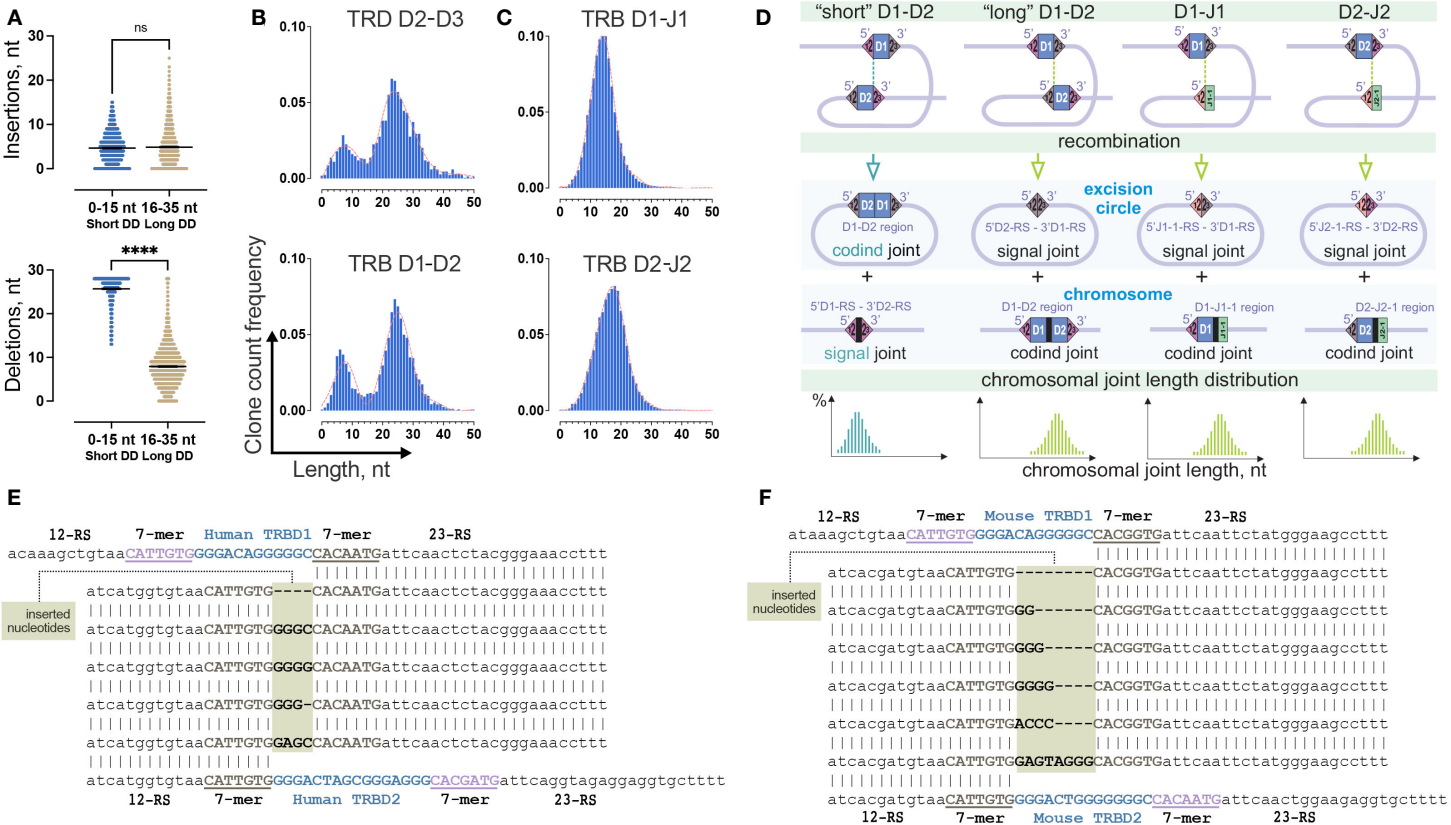
Two-site TRB D-D recombination as a source of bimodality in D-D junction length distribution. **(A)** Comparison of the number of inserted nontemplate and deleted nucleotides in groups of “short” and “long” TRB D-D rearrangements. Bars and whiskers represent the mean with a 95% confidence interval. **(B)** Comparison of length distribution patterns of TRB D-D and TRD D-D rearrangements. Pooled 15 samples are displayed. Length calculation performed excluding RS. **(C)** Length distribution of TRB D-J rearrangements. Pooled 15 samples are shown. **(D)** Formation of coding and signal joints during recombination between D1 and D2 genes and D and J genes in the TRB locus. **(E)** Inserted nucleotides in human TRB 5’D2-RS – 3’D1-RS signal joints from excision circle DNA. **(F)** Inserted nucleotides in mouse TRB 5’D2-RS – 3’D1-RS signal joints from excision circle DNA. P values less than 0.0001

The TRB D-D junction lengths in the two observed peaks had a substantial difference of ~20 nucleotides. “Long” rearrangements contain joined TRBD1 and TRBD2 coding regions. “Short” rearrangements lack identifiable coding D-regions (**Figure 2A**). There are at least two potential explanations for the observed “short” D-D rearrangements: it could be a result of the lower activity of TdT and/or higher activity of exonuclease in some portion of thymocytes, or it could represent intrachromosomal 5’D1-RS - 3’D2-RS signal joints as proposed for similar rearrangements in mice (9). To resolve this issue, we compared the numbers of deleted and inserted nontemplated nucleotides in TRB D-D rearrangements (**Figure 4A**). We detected no difference in the number of inserted nucleotides (p=0.114, two-tailed Mann-Whitney test), which indicates that the difference in lengths of two observed types of TRB D-D rearrangements is based solely on missing D-regions (p<0.0001, two-tailed Mann-Whitney test).

To better understand whether this bimodal distribution is unique across the V-D-J rearrangements, we similarly analyzed partial TRB D1-J1, and D2-J2 rearrangement junctions, which do not contain D-D junctions. Additionally, we analyzed completely different locus – T-cell receptor delta (TRD) chains, which contain three D genes and produce the only conventional partial D-D rearrangements, TRDD2-TRDD3. The distribution of the D-J junction length has a single peak (**Figure 4C**) in contrast to TRD D-D, which shows a bimodal distribution similar to TRB D-D (**Figure 4B**). Thus, the observed bimodal distribution is most likely characteristic of D-D rearrangements. The main structural similarity of D-D pairs of TRB and TRD loci is the presence of two putative sites of recombination (two 12/23 RS) in contrast to TRB D-J pairs, which only have a single site (one 12/23 RS) (**Figure 4D**). Therefore, the probable reason for the bimodal distribution is the formation of two different TRB D-D RS synapses with corresponding chromosomal DNA cleavage in two possible positions, (i) with the inclusion of D1 and D2 region sequences in the TRB D-D rearrangement (resulting in a D1-D2 coding joint on the chromosome and a 5’D2-RS – 3’D1-RS signal joint on an excision circle) or (ii) the exclusion of the D1 and D2 region sequences (resulting in a 5’D1-RS – 3’D2-RS signal joint on the chromosome, and a D1-D2 coding joint eliminated on an excision circle (**Figure 4D**).

### Excision circle 5’D2-RS – 3’D1-RS signal joints

To understand whether nontemplate nucleotides can be present in the signal joints, in addition to capillary sequencing (**Figure 2B**) we performed high-throughput sequencing of the amplicon of human 5’D2-RS – 3’D1-RS signal joints from excision DNA circles. Providing single molecule resolution, it allows the detection of not only major signal joint variants, as does capillary sequencing but also minor ones. The results clearly show that other noncanonical signal joint structures were present at a frequency 10 times less than in classical signal joints previously analyzed. These secondary signal joints appeared to have unexpected structures (**Figure 4E**). The difference between these and the classical ones is the presence of several additional nucleotides between RS, which only partially match the distal nucleotides of the D1 and D2 genes. Similar secondary signal joints with additional nucleotide insertions were detected in a mouse (**Figure 4F**). Thus, following the exact 12/23 rule, “short” D-D rearrangements are produced as a result of signal joint formation rather than typical coding joint formation. In this case, the existence of excised coding TRBD1 and TRBD2 gene joints is expected (**Figure 4D**). However, we could not detect it in our excision circles sequencing data.

Both “short” and “long” TRB D-D rearrangements, observed in mature T cells similarly to D-J rearrangements, remain in their partial state presumably due to a lack of further processing. It is also possible that they represent a significant percentage of the allelically excluded TRB in some mature T cells.

### D1-D2 coding joint contribute to productive V-D1-D2-J2 chains

**Figure 5:**
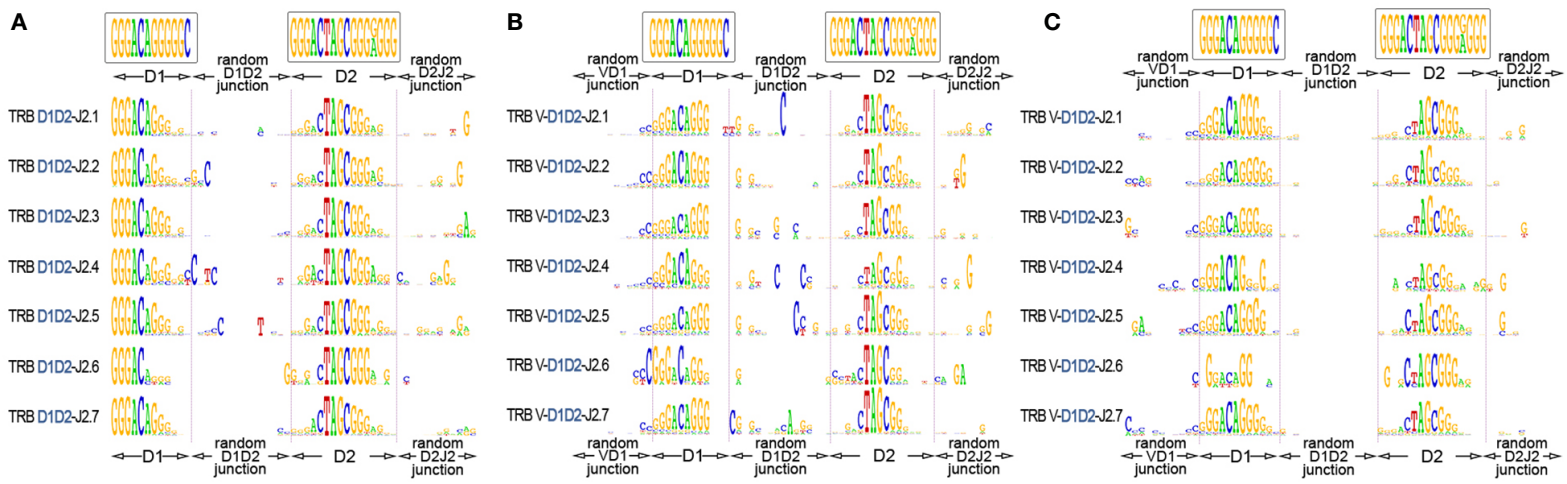
TRB D1-D2 junctions in the D1-J2 and V-D-J2 rearrangement structures. **(A)** Logo diagram of TRB D1-D2-J2 junctions among bulk PBMCs (15 donors, data were generated in this study). **(B)** Logo diagram of TRB V-D1-D2-J2 junctions among bulk PBMCs (11 donors from previous research (17), data: PRJNA847436). **(C)** Logo diagram of TRB V-D1-D2-J2 junctions among memory (CD45RO) T cells. (11 donors from previous research (17), data: PRJNA847436). The results are displayed for pooled samples.

The “long” TRB D-D rearrangements carry fully intact RS, which allows them theoretically to recombine further with J genes using 3’D2-RS (23-RS) and then with V genes using 5’D1-RS (12-RS). To investigate this possibility, we identified TRB D-D junctions in partial TRB D-J rearrangements (**Figure 5A**) and then in complete productive V-D-J rearrangements (**Figure 5B**) from bulk PBMC of 15 and 11 healthy donors respectively. TRB D-J was analyzed at the DNA level. TRB V-D-J was analyzed at the RNA transcript level to additionally verify the functionality of searched in-frame V-D-D-J rearrangements. The results unambiguously show the simultaneous presence of both TRBD1 and TRBD2 regions in partial D-J and complete V-D-J rearrangements. The most conservative estimation (7-mers of D1 and D2) of D1-D2-J2 in total D-J2 fraction was 8.4%, and that of V-D1-D2-J2 in the productive V-D-J2 fraction was 0.15% for bulk T cells.

Finally, we performed the same analysis for memory (CD45RO) T cells to understand whether V-D-D-J rearrangement-derived TRB chains are genuinely productive and are thus capable of recognizing epitopes similar to conventional TCRs. We successfully identified D1-D2 regions in the structures of complete productive TCR V-D-J rearrangements in memory T cells of 15 individuals (**Figure 5C**) at the level of 0.11% of the total functional V-D-J2 fraction. This result shows that TCR genes containing D1-D2 regions have a functional impact on adaptive immunity with a capacity for antigen recognition.

## Discussion

Formation of a functional adaptive immune system includes immune receptor diversity generation via V-D-J recombination and subsequent negative and positive clonal selection waves in the thymus and second lymphoid organs. In this study, we confirmed that the D1-D2 coding joint product of V-D-J recombination of the TRB locus contributes to the human functional immune repertoire. The existence of this process was confirmed in rats and mice. Our findings suggest that D-D rearrangements in TRB locus are not rare aberrant events but rather a common part of TRB formation and diversification processes in mammals.

Our data provide evidence that the use of two different recombination sites, at this stage, produces two groups of partial D-D rearrangements: “short” and “long”. The “short” ones contain a signal joint 5’D1RS – 3’D2RS and may be followed by a direct V-J2 rearrangement leading to TRB chains which lack D regions. The “long” ones contain a D1-D2 coding joint which may rearrange further to form a partial D1-D2-J2, product and then a complete V-D1-D2-J2 rearrangement expressing a TRB chain with two D-regions. Thus both “short” V-J2 and “long” V-D1-D2-J chains contribute to the overall TRB combinatorial V-(D)-J diversity.

Deleting J(1-6)-C1 cluster during D-D recombination mechanically increases the frequency of J2 genes in TRB generation. This new knowledge is the basis for the further improvement of the well-established TRB generation models (18, 19), which has been successfully applied to the computational evaluation of TR specificity (20, 21).

The “short” D-D group cannot be traced directly into complete TRB since they do not have recognizable parts of D genes. At this point, we can only hypothesize that they may contribute to shorter TRB fractions which are reported to participate in the immune response to common infections and are abundant and public (22, 23). The observed skewing of J gene usage toward the J2 cluster in complete D-region-free TRB rearrangements aligns with this hypothesis (**Supplementary Figure 1**). The “short” D-D rearrangements most likely just facilitate direct V-J recombination although we cannot exclude the possibility that the short D-D region with nongenomic inserted nucleotides acts as a surrogate single D region in V-D-J recombination.

The traces of the “long” group can be partially detected in partial and complete TRB rearrangements. Meanwhile, the “long” fraction is clearly contributing to antigen recognition being directly detected in memory T-cell subsets in this study.

In summary, this study analyzed an almost unnoticed component of the TCR diversity generation process. While we do not completely understand the extent to which it affects T-cell immunity in health and disease, we have clearly established it’s existence. We believe this newly identified rearrangement profile is significant for both clinical and basic scientific knowledge. Partial TRB D-D rearrangements can be applied as a novel marker for monitoring minimal residual disease (24, 25), clonal analysis in lymphoid malignancies (26, 27) and normal lymphocytes to better understand how the immune system generates the magnitude of diversity.

## Methods

### Sample collection and DNA isolation

The research was conducted according to the Declaration of Helsinki. All human subjects gave standard informed consent. The study was approved by the local ethical committee at Pirogov Russian National Research Medical University. In this study, we used DNA extracted from peripheral blood mononuclear cells (PBMC) from 15 healthy human individuals with the age range of 30-50 years, one wild-type mouse (ICR strain, 8 weeks, female) and one wild-type rat (SD strain, 10 weeks, female); DNA from the thymus of one human donor (16^th^ donor, dissection of the thymus was a standard part of the heart surgery), DNA from thymi of the same mouse, and the same rat (**Figure 1A**); DNA from bulk CD8+ and bulk CD4+ human T cells. DNA from the RMS cell line and CD19+ human B cell fraction were used as a negative control.

### Isolation of PBMC and lymphocyte fraction

Peripheral blood mononuclear cells were isolated by Ficoll-Paque (Paneco, Russia) density gradient centrifugation according to manufacturer’s protocol. Bulk CD4+ and CD8+ T cells and CD19+ B cells were isolated from PBMC suspension using a magnetic separation approach with Dynabeads Positive Isolation Kits (Invitrogen, USA). DNA from all collected samples was isolated by FlexiGene DNA kit (Qiagen, Germany).

### TRB D1-D2 and D-J library preparation and sequencing

The library for high-throughput sequencing was obtained in two sequential PCR reactions (28). The first (target) 25 µl PCR contained 100 ng DNA isolated from PBMC, thymus or T-cell subsets, 1X Turbo buffer, five units of HS Taq polymerase, 200 µM of each dNTP (all Evrogen, Russia), and 0.2 µM of each primer specific to genomic flanks of TRB D1-D2 junction for DD library (**Figure 1B**), primers flanking D and J genes for D-J library and primers flanking D2 and D3 for TRD D2-D3 library (all MiLaboratories, USA). The amplification profile was 94°C for 3 min, followed by 10 cycles of 94°C for 20 sec, 57°C for 90 sec, and 72°C for 40 sec, followed by an additional 15 cycles of 94°C for 20 sec and 72°C for 90 sec. All PCR cycles were performed with Ramp 0.5°C/sec. Six replicates were obtained for each PBMC and thymus sample to reach the average sample size equal to ~100,000 analyzing cells (600 ng input DNA total per sample).

Obtained amplicons were purified using 1X AmPure XP beads (Beckman Coulter) and used as a template for the second PCR. The second (indexing) 25 µl PCR contained 1X Turbo buffer, 2.5 units of HS Taq polymerase, 200 µM of each dNTP, and 1 µl of Unique dual indexes primers (Illumina, USA). The amplification profile was as follows: 94°C for 3 min, followed by 15 cycles of 94°C for 20 sec, 57°C for 20 sec, and 72°C for 40 sec. Obtained amplicons were purified using 0.8X AmPure XP beads (Beckman Coulter, USA), pooled, and sequenced on Illumina NextSeq or MiSeq machine with coverage ~100,000 reads per sample (**Figure 1C**).

### TRB D1-D2 excised signal joint identification

The signal joint detection was performed using PCR with subsequent capillary sequencing. To obtain the target PCR product, 150 ng of DNA isolated from the thymus (human, rat, and mouse) was used as a template in a 25 µl PCR reaction containing 1X Turbo buffer, 2.5 units of HS Taq polymerase, 200 µM of each dNTP and 0.2 µM of each primer specific to signal joint flanks (**Figures 1B**, **2B**; **Table 1**).

**Table 1:** Primers for excised signal joint detection.

Species	Dir primer sequence	Length	Rev primer sequence	Length
Human	5’-ACCCAGGAGGAAAGAAGAGGACT-3’	23	5’-GTGATGCATGTTCCAAGGAGGG-3’	22
Rat	5’-TTGTAAAGGTTTCCCATAGAATTG-3’	24	5’-AGGGGAAACCCAGTGACATAG-3’	21
Mouse	5’-GTAAAGGCTTCCCATAGAAT-3’	20	5’-TGATATAGATGTTCTCCCAG-3’	20

Dir primer sequence: located upstream (5’) of TRB 5’D2-RS. Rev primer sequence: located downstream (3’) of TRB 3’D1-RS.

The amplification profile was 94°C for 3 min, followed by 45 cycles of 20 sec of 94°C, 20 sec of 58°C and 40 sec of 72°C. Signal joints for each analyzed species were amplified in separate PCR. Capillary sequences of the target PCR products were obtained as a service (Evrogen, Russia).

### TRB D1-D2 and D-J repertoire reconstruction and analysis

TRB D-D, D-J, and TRD D-D rearrangements were extracted from fastq files using MiXCR 3.0 software (29) with additional genomic reference, containing TRD D2, D3, TRB D1, D2, and J genes with their genomic flanks from IMGT database (4). VDJtools software (30) was used for subsequent post-analysis: clonotype diversity calculation, clonotype uniqueness analysis, and detection of inserted and deleted nucleotides in the D-D junction. Statistical analysis and plotting were performed using GraphPad Prism 9.0. To detect D-D-J rearrangements, the standard terminal “grep” function was used to search for 7 nt k-mers (ggactag, gactagc, actagcg, ctagcgg, tagcggg, agcgggg, agcggga) specific for D2 genes in D1-J2 junction fragment. The sequences containing target k-mers were subjected to multiple alignment using SnapGene software with MAFFT v7.471 algorithm (31). The LOGO diagrams were produced by WebLogo software (32) using aligned sequences for D-D-J and non-aligned ones for D-D rearrangements.

### V-D-D-J rearrangements detection

TRB V-D-J rearrangements were extracted from previously published deep TCR-seq dataset (17) for bulk PBMCs of 11 healthy donors (24-60 years old) before vaccination (PRJNA847436) using MiXCR 3.0 with default parameters. Obtained clonotype tables were converted into VDJtools format using “Convert” function with “-S mixcr” parameter. The level of quantitative bias was checked using iROAR software (33). The TRBJ2 containing clonotypes with detected D1 region were extracted from the analyzed dataset using “FilterBySegment” function and then were separated to functional and nonfunctional clonotype tables using the function “FilterNonFunctional”. The final detection, aligning, and displaying of V-D-D-J2 rearrangements were performed as described above for D-D-J2 rearrangements.

## Data availability statement

The datasets presented in this study can be found in online repositories. The names of the repository/repositories and accession number(s) can be found below: PRJNA952099 (SRA).

## Ethics statement

The studies involving humans were approved by the Pirogov Russian National Research Medical University local ethics committee. The studies were conducted in accordance with the local legislation and institutional requirements. The human samples used in this study were gifted by another research group. Written informed consent for participation was not required from the participants or the participants' legal guardians/next of kin in accordance with the national legislation and institutional requirements. The use of animals was approved by local committee for the control of the keeping and use of laboratory animals.

## Author contributions

AK: the idea, study design, funding, data analysis, manuscript drafting. AS, AM, LB and IK: performed the experiments and data analysis. YL, IM, DC: resources, samples, mentorship, manuscript drafting. All authors contributed to the article and approved the submitted version.

## Supplementary material

Supplementary Figure 1 Comparison of TRBJ1 and TRBJ2 containing clonotypes number among complete TRB rearrangements lack coding D-regions. **(A)** Nonfunctional (out-of-frame) clonotypes. **(B)** Functional (in-frame) clonotypes. Samples: PBMC from 11 healthy donors in two replicates (PRJNA847436). Wilcoxon two-tailed signed rank test.
