# Supplementary figures and images for: Novel bimodal TRBD1-TRBD2 rearrangements with dual or absent D-region contribute to TRB V-(D)-J combinatorial diversity
Source: Front Immunol. 2023 Sep 7;14:1245175. doi: 10.3389/fimmu.2023.1245175 (PMC10513440; doi:10.3389/fimmu.2023.1245175)

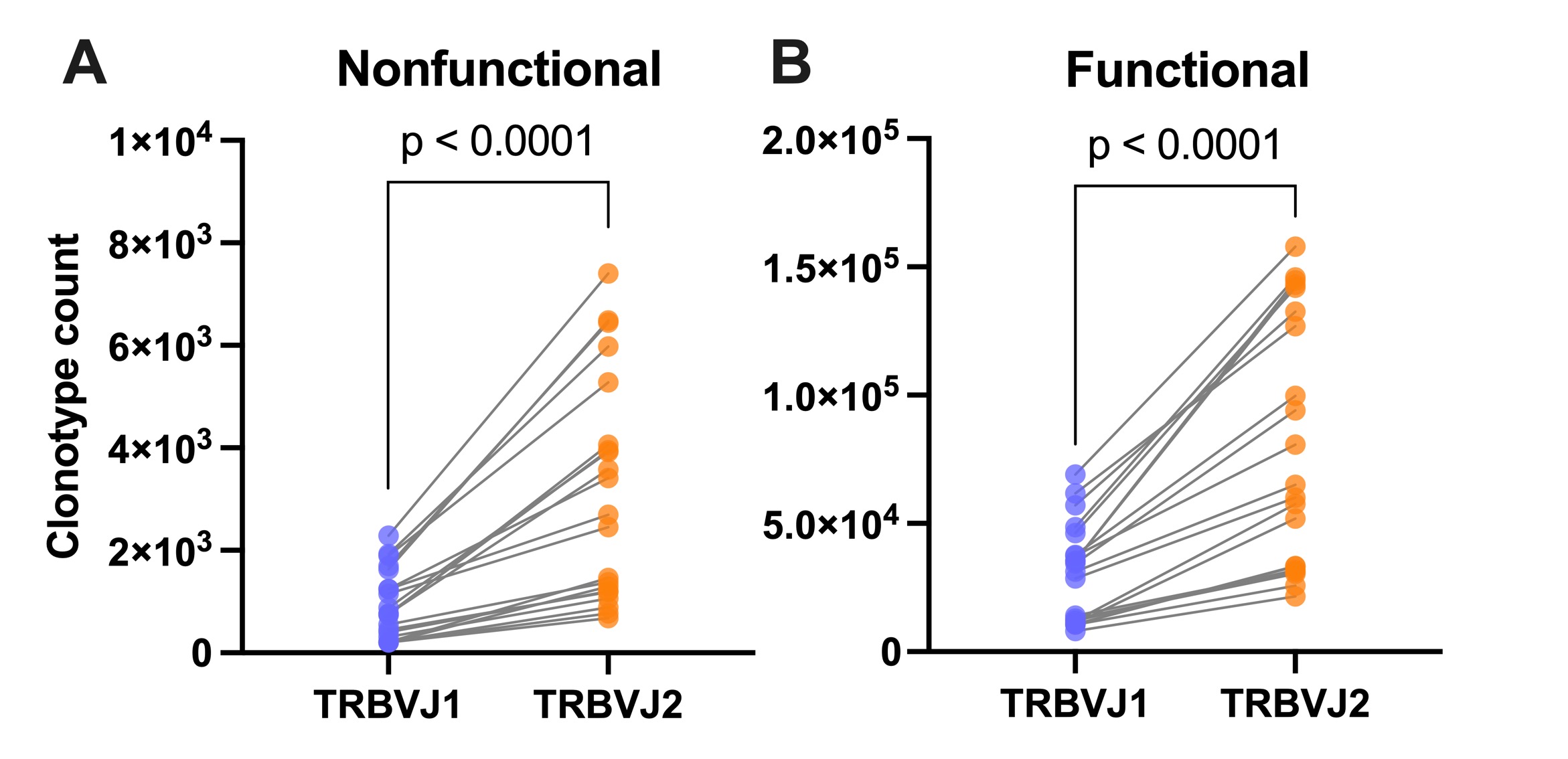

Supplement: Supplementary Figure 1 — Comparison of TRBJ1 and TRBJ2 containing clonotypes number among complete TRB rearrangements lack coding D-regions. (A) Nonfunctional (out-of-frame) clonotypes. (B) Functional (in-frame) clonotypes. Samples: PBMC from 11 healthy donors in two replicates (PRJNA847436). Wilcoxon two-tailed signed rank test. [file Image_1.jpeg]
